# Detecting Task Difficulty of Learners in Colonoscopy: Evidence from Eye-Tracking

**DOI:** 10.16910/jemr.14.2.5

**Published:** 2021-07-13

**Authors:** Liu Xin, Zheng Bin, Duan Xiaoqin, He Wenjing, Li Yuandong, Zhao Jinyu, Zhao Chen, Wang Lin

**Affiliations:** School of Computer and Communication Engineering, University of Science and Technology Beijing, Beijing, China; Surgical Simulation Research Lab, Department of Surgery, University of Alberta, Edmonton, Alberta, Canada; Department of Rehabilitation Medicine, Jilin University Second Hospital, Changchun, Jilin, China; Department of Surgery, University of Manitoba, Winnipeg, Manitoba, Canada; Department of Surgery, Shanxi Bethune Hospital, Taiyuan, Shanxi, China; Beijing Key Laboratory of Knowledge Engineering for Materials Science, Beijing, China; *

**Keywords:** colonoscopy, simulation, eye-tracking, navigation, Deep Convolutional Generative Adversarial Networks (DCGANs), Long Short-Term Memory (LSTM)

## Abstract

Eye-tracking can help decode the intricate control mechanism in human performance. In healthcare, physicians-in-training require extensive practice to improve their healthcare skills. When a trainee encounters any difficulty in the practice, they will need feedback from experts to improve their performance. Personal feedback is time-consuming and subjected to bias. In this study, we tracked the eye movements of trainees during their colonoscopic performance in simulation. We examined changes in eye movement behavior during the moments of navigation loss (MNL), a signature sign for task difficulty during colonoscopy, and tested whether deep learning algorithms can detect the MNL by feeding data from eye-tracking. Human eye gaze and pupil characteristics were learned and verified by the deep convolutional generative adversarial networks (DCGANs); the generated data were fed to the Long Short-Term Memory (LSTM) networks with three different data feeding strategies to classify MNLs from the entire colonoscopic procedure. Outputs from deep learning were compared to the expert’s judgment on the MNLs based on colonoscopic videos. The best classification outcome was achieved when we fed human eye data with 1000 synthesized eye data, where accuracy (91.80%), sensitivity (90.91%), and specificity (94.12%) were optimized. This study built an important foundation for our work of developing an education system for training healthcare skills using simulation.

## Introduction

Each year in Canada, about 970,000 colonoscopies are performed (9). During the examination, the physician (endoscopist) inserts a long but flexible tube carrying a camera (colonoscope) into the patient’s lower gastrointestinal (GI) tract to detect any abnormality (1). Colonoscopy is often a painless procedure that can benefit people with cancer screens. However, physicians-in-training (endoscopists) require extensive practice to gain the necessary skills in manipulating the scope for completing the inspection and basic surgical procedures, such as taking a tissue sample (biopsies) or removing a polyp (polypectomy) (15) (5). Medical simulation has been widely used in recent years to give trainees abundant opportunities to reach their proficiency (10; 14 ; 30).

In any simulation-based training session, faculty members are needed for observing the performance and providing concrete feedback to trainees (29). Feedback and instructional message are important for skill improvement especially when trainees encounter any task difficulty (4). Education outcomes will be questionable if trainees are deprived of feedback on their performance (8). When it comes to a large training group, the teaching burden on faculty members will be dramatically high (7). Personal feedback also comes with inherited drawbacks, including inconsistency and bias (22). As we are entering the era of artificial intelligence (AI), we ought to create an education system that can release the burden of faculty members while providing consistent teaching feedback to trainees.

The initial step to achieve the above goal is to spot the moment of task difficulty during trainees’ performance. Once a moment of task difficulty is detected, we then need to figure out a way to deliver an appropriate instructional message to the trainee in the applicable format. The latter step can be achieved using a new interface, such as augmented reality (17). In this paper, we focus on the first step, which is how to detect the moment of task difficulty based on trainees’ behavioral data.

In this study, we report our effort on detecting task difficulty using trainees’ eye-tracking data. Specifically, we record trainees’ eye movements while they are performing a colonoscopic procedure. During a colonoscopic procedure, the scope is navigating inside the GI tract. The interior structure of the colon is alike in all directions; trainees often do not have sufficient visual cues for guiding their scope movement (28). Adding to the difficulty, the direction of the colonoscope is controlled by two-wheeling knobs in the hands of the trainee, which are difficult to manipulate. The reduced visual cues and the difficulty in controlling scope direction can lead to a moment of navigation loss (MNL), where global spatial and local anatomic references are confusing to an operator (31). When this happens, effective inspection and manipulation are suspended. In the worst scenario, the tip of the scope may push the wall of the colon with an exceeded amount of force, causing severe complications including bleeding and perforation of the colon (18; 21; 24). In any training session of colonoscopy, the MNL is a sign of dangerous maneuver calling immediate assistance and guidance. Our training instructor will step in at the MNL to help the trainee to regain navigation before an undesirable consequence occurs.

In this project, we investigated whether the eye-tracking signals can give us enough evidence to detect the MNL accurately during a colonoscopic procedure. Specifically, we analyzed eye gaze and pupil dilation characteristics and applied AI to help us find MNLs during colonoscopy. To achieve this goal, we first manually annotated those MNLs recorded in colonoscopic videos. We then compared a list of eye-tracking measures including fixation and saccade between the MNL and non-MNL (the moment without navigation loss). Deep learning (DL) algorithms were applied to those data for detecting the MNL during the entire colonoscopic procedure. DL outcomes on MNLs were eventually compared to expert’s judgment based on colonoscopic videos. We hypothesized that the deep learning algorithm will achieve an outcome (accuracy, sensitivity, and specificity > 90%) in spotting the MNL during a colonoscopic procedure.

## Methods

### Participants

The study was conducted at the Surgical Simulation Research Lab of the University of Alberta. Ten junior surgical residents and university students at the University of Alberta were recruited. They were in the early surgical training phase with zero to less than 10 opportunities to practice colonoscopic procedures. The study was reviewed and approved by the Health Research Ethical Board of the University of Alberta. Written consent was obtained from each participant before entering the study.

### Tasks

**Figure 1.**
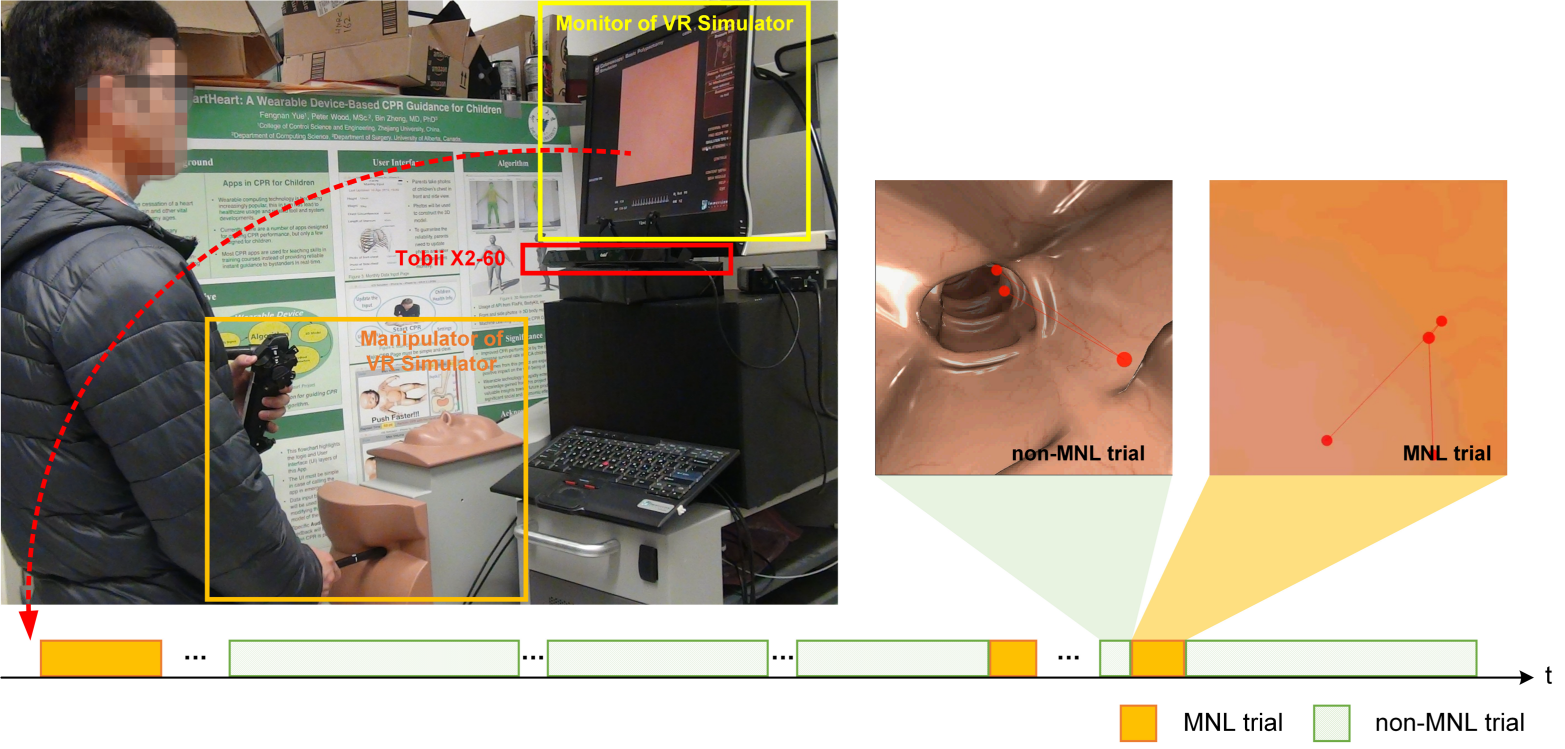
Experimental setup. A Tobii X2-60 was installed under the monitor of the Accutouch VR Endoscopic Simulator. Throughout a colonoscopic procedure, a moment of navigation loss (MNL) might occur several times (bottom panel, highlighted in light yellow). During a MNL, the lumen of the colon disappeared; the participant’s eye was scanning the wall of the colon to search for the correct direction.

Participants were asked to perform colonoscopic cases on the Accutouch Colonoscopic Virtual Reality Simulator (Figure 1. CAE Healthcare, Montreal, Quebec**)**. The simulated case was a 54-year-old female patient who has two polyps in the ascending colon that need to be inspected with colonoscopy. The participant was instructed to visualize the ileocecal valve and take pictures of the two polyps. Each participant practiced for three minutes to familiarize with the simulation and scenario before the trial. Specific feedback for colonoscopic performance was not provided once the trial started.

### Data Collection

When the participant was performing the colonoscopic procedures, the simulator recorded the entire performance video from the scope view. These *scope videos* were used for inspecting the MNL. At the same time, an eye-tracker (Tobii X2-60, Tobii Technology, Danderyd, Sweden) attached to the bottom of the endoscopic monitor recorded the participant’s eye movement. The sampling frequency of Tobii X2-60 is 60 Hz and the recording resolution of the video is 1024 × 768 (in pixel). Specialized software (Tobii Studio 3.3.2, 2017) was used to extract eye movement measures for further analysis.

### Data Extraction

Colonoscopic videos were replayed frame-by-frame to label the MNLs by an experienced endoscopist. In this study, a MNL started at the moment of scope pointing to the wall of the colon, leaving the bowel lumen completely disappeared from the scope view. Once the bowel lumen re-appeared, the MNL ended and the non-MNL began. The duration of each MNL and non-MNL was recorded. Data acquired from eight subjects were used for statistical analysis and for training the deep learning algorithm. From their colonoscopic video, a total of 51 MNLs and 77 non-MNLs were annotated by an experienced endoscopist. After training, verification was performed on data collected with another two participants differing from the training dataset, where 17 MNLs and 44 non-MNLs were annotated by an experienced endoscopist.

From the eye-tracking, Tobii studio reported three different types of eye movements, saccade, fixation, and pupil dilation. The fixation is based on the I-VT (Velocity-Threshold Identification) fixation filter in Tobii studio (27). The velocity feature is calculated by measuring the distance between two adjacent data points. Here, the velocity threshold is set to 2.1 pixels/ms. Each point is then labeled as a fixation if the velocity is below a certain threshold or otherwise as a saccade. The minimum fixation duration is set to 90 ms. The fixations duration below 90 ms will be discarded from the analysis.

#### Eye gaze measures

The eye gaze parameters included saccade/fixation number (#), saccade duration (s), saccade/fixation frequency (#/s), and percentage of saccade duration were reported for MNL and non-MNL respectively. Significant statistical tests (see Table 1) on these measures over MNL and non-MNL were performed, and the results were used for selecting the key features to feed the machine learning algorithms.

#### Saccadic amplitude

When falling into a MNL, a subject might be more actively searching for visual cues to regain their navigation, which can be described as the saccadic amplitude (SacAmp, the angular distance between two succeeding points of momentary stop in a translational plane). We calculated the cumulative frequency of SacAmp above 1.5, 2.0, 2.5, 3.0, 3.5, 4.0, 4.5, 5.0, 5.5, 6.0, 6.5, 7.0, and 7.5 degrees for MNL and non-MNL respectively. The SacAmp range where the largest difference was observed between MNL and non-MNL was used for feeding the deep learning algorithms.

#### Euclidean distance between fixations

In addition to the SacAmp, we used the *fixation distance* (FixDis, the Euclidean distance in pixels between two fixations) to describe the searching behavior during an MNL. We calculated the cumulative frequency of FixDis below 25, 50, 75, and 100 pixels for MNL and non-MNL respectively. The FixDis range where the largest difference was observed between MNL and non-MNL was used for feeding the deep learning algorithms.

#### Pupil size

A subject’s pupil size may enlarge during a MNL as their level of stress increases (35). The pupil size during the colonoscopic procedure was affected by many factors and displayed enormous individual differences. To make pupillary data comparable, we introduce a term called the adjusted pupil size (APS). We first found the minimum and maximum value of pupil diameter from each subject during the entire colonoscopic trial and then calculate the APS at any given time using the equation below.

(1) $$\text{APS}=\frac{ps-{\text{ps}}_{\min}}{{\text{ps}}_{\max}-{\text{ps}}_{\min}}\times 100\%$$

Here, ${\text{ps}}_{\max}$ and ${\text{ps}}_{\min}$ are the maximum and minimum pupil sizes during the colonoscopic procedure, where ps is the current pupil size.

Please note the APS is reported in percentage; it means, subject’s pupil size at any given time is reported as the percentage to the range of pupil change over the entire trial. We calculated APS within the fixation (APS_fix_) and saccade (APS_sac_) phase as well as in the entire trial (APS_trial_) and compared them between MNL and non-MNL.

We divided the range of APS_trial_ (0-100%) equally into 20 intervals with an index from 1 to 20 (e.g. the index 1 represents a 0-5% APS_trial_ change) to find the index where the APS_trial_ has the largest difference between MNL and non-MNL. We also compared the cumulative frequency of APS_trial_ between 55% and 100% in MNL and non-MNL.

### Statistical Analysis

Statistical analysis was used to determine which measures could better identify MNLs. We hypothesized that measures showing a significant difference between MNLs and non-MNLs would be good features for training the algorithms. Statistical analysis was performed using SPSS 25.0 (IBM Corp, Chicago, USA). The Shapiro-Wilk’s test (p > 0.05) and a visual inspection of histograms and normal Q-Q plots (Quantile-Quantile plots) showed that eye fixation, saccade, and pupil data were approximately normally distributed. The Independent Samples t-Test was run to compare eye measures between MNL and non-MNL. Means and standard errors were reported for significance, with a priori level of 0.05 (Table 1, 2, and 3).

### Deep Learning

#### Feature selection

We selected the significantly different measures from time and frequency in saccade and fixation, saccade amplitude, fixation distance, and pupil measures. We noted that a growing amount of data will dramatically increase the computation load on the computer. Our goal is to achieve the best learning outcome with a moderate amount of data.

#### Training process

The training process included two stages. In the first stage, we trained *DCGANs* (Deep Convolutional Generative Adversarial Networks) algorithm using eye data from human subjects to learn their probability distribution and for augmenting eye data by computer (25). Generative adversarial networks are a class of machine learning frameworks including two neural networks. These two neural networks contest with each other in a zero-sum game, where one agent’s gain is another agent’s loss. Computer synthesis was a necessary step for this study as human data was not enough. DCGANs can explore the potential pattern from complex data and augment high-quality synthesized samples supplementing the training sets for intelligent detections (25). There were many pieces of research using DCGANs in medical data augmentation, such as voiceprint samples augmentation for Parkinson (34), CT image reconstruction for skin and liver lesion classification (2; 23), Chest X-Ray pathology synthesizing (26), et al. It can be considered as a valid method for data augmentation.

**Figure 2.**
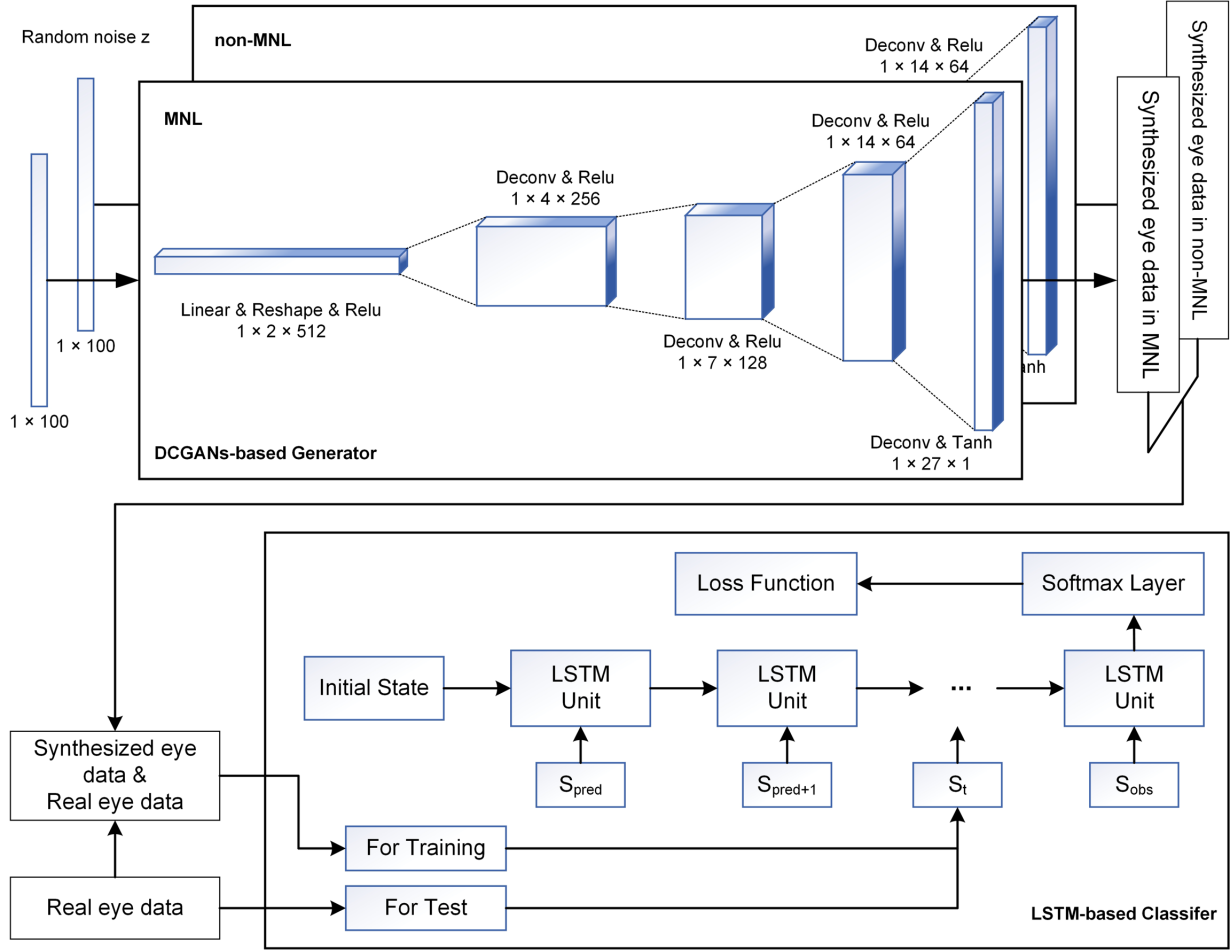
Illustration of AI architecture for detecting MNLs during colonoscopy. Top Panel: DCGANs-based generator for synthesizing data for MNL and non-MNL phases independently. Bottom Panel: flowchart of feeding data to LSTM model to detect and classify MNLs in a colonoscopic procedure.

As shown in Figure 2 (Top Panel), the DCGANs were trained for non-MNL and MNL independently, so the label of synthesized eye data was known. Specifically, the number of training epochs in the DCGANS was set to 5000 with a batch size of 32. The variable learning rate was 0.001 and the momentum term was 0.5 for the Adam optimizer. The a priori input noise variables were a 100-dimensional vector with uniform randomly synthesized values in [-1,1]. The dimension of filters in the first convolutional layer was 5×5×64 and the number of units for the fully connected layer was set to 1024 for both generator and discriminator networks.

To verify the eye data synthesized by DCGANs, we constructed a probability distribution of synthesized data points over the real human data and created a low-dimension map for visual inspection using the *t-SNE* (t-distributed Stochastic Neighbor Embedding) algorithm (32).

The second stage of the training process was using the real and synthesized eye data to train the *LSTM* (Long Short-Term Memory networks) detection model as shown in Figure 2 (Bottom Panel). The LSTM unit is composed of a cell, an input gate, an output gate, and a forgetting gate (16). The cell memorizes values over arbitrary time intervals and the three gates regulate the flow of information into and out of the cell. This unit setting is well-suited to solve the vanishing gradient problem in deep networks. Furthermore, LSTM is a powerful computer algorithm for classifying human data (13; 20; 33; 36). It can process single data points as well as an entire-time series. The number of training epochs for LSTM was set to 1000 and the batch size was 32. The number of units for each layer was set to 32 and the time step (including a feature vector) was set to 1. The weights were initialized with the orthogonal matrix and the offset terms were initialized to 0. The softmax layer had two nodes: MNL and non-MNL. The categorical cross-entropy loss function adopted the Root Mean Square Prop algorithm for optimization. The hyper-parameters of the deep learning model were selected according to the reference (16; 25) and determined by many empirical trials.

#### Validation

We input phases of 5 seconds for AI detection. Eye metrics during these phases (5-second windows), including features of eye movement data, were fed to LSTM for classifying the MNL and non-MNL. Here, we compare the LSTM outcomes to human judgment on MNL and non-MNL. Specifically, the accuracy, sensitivity, and specificity of LSTM outcomes were reported for three data feeding strategies. We also examined the improvement of classification outcomes after adding computer synthesizing data. In this paper, we reported outcomes using human data only, human data plus 200 synthesized data points, and human data plus 1000 synthesized data points.

## Results

### Eye Gaze Difference

#### Time and frequency in saccade and fixation

Table 1 shows the comparison between time and frequency measures of eye movement during MNLs and during non-MNLs. The MNL had a significantly shorter duration than non-MNL. The saccade durations in MNL were significantly shorter than in non-MNL. The saccade and fixation number in MNL was significantly less than in non-MNL.

#### Saccade amplitude

Table 2 shows the comparison between saccade amplitude during MNLs and non-MNLs. The saccade amplitude was significantly larger in MNL than in non-MNL. The largest difference between the cumulative frequency of saccadic amplitude in NML and non-NML was observed when the saccadic amplitude was above 2.5 degrees (highlighted in bold in Table 2).

**Table 1.** Time and frequency of saccade and fixation compared between MNL and non-MNL.

**Parameters**	**MNL**	**non-MNL**	**P-value**
	***Mean ± SE***	***Mean ± SE***	
phase duration (s)	12.53 ± 1.45	22.97 ± 2.01	**< 0.001**
saccade duration (s)	5.49 ± 1.00	10.78 ± 1.44	**0.003**
saccade number	52.06 ± 7.91	101.19 ± 9.36	**< 0.001**
fixation number	23.69 ± 2.77	40.94 ± 3.89	**< 0.001**
saccade frequency (#/s)	4.29 ± 0.26	4.60 ± 0.18	0.329
fixation frequency (#/s)	2.04 ± 0.08	1.95 ± 0.08	0.406
gaze event frequency (#/s)	6.34 ± 0.28	6.55 ± 0.20	0.530
mean duration of saccade for each time (s)	0.10 ± 0.00	0.09 ± 0.00	0.474
mean duration of fixation for each time (s)	0.30 ± 0.02	0.28 ± 0.01	0.490
saccade duration percent (%)	41.19 ± 3.07	44.10 ± 2.91	0.506
saccade number percent (%)	65.36 ± 1.51	68.91 ± 1.31	0.082
fixation number percent (%)	34.64 ± 1.51	31.09 ± 1.31	0.082

#### Fixation distance

**Figure 3.**
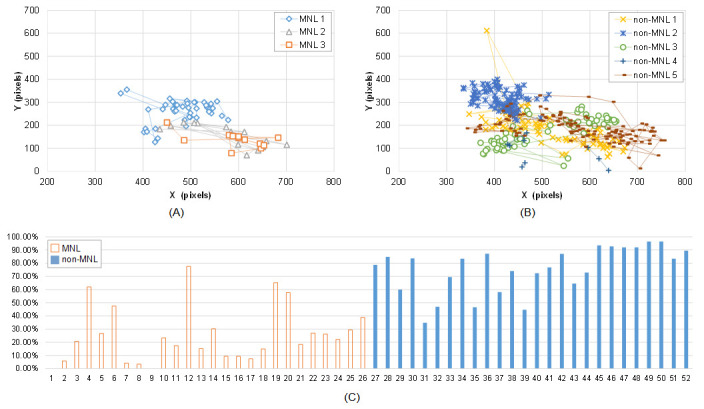
Fixation distance and adjusted pupil size in MNL and non-MNL. A-B): Subject 1’s fixation trajectory in MNL and non-MNL during a colonoscopy; C) Subject 5’s cumulative frequency of APS in the range of [55%, 100%]. From 1 to 26 (horizontal axis) are the cumulative frequency in 13 MNL phases; from 27 to 52 (horizontal axis) are the cumulative frequency in 13 non-MNL phases. Here, an odd number in the horizontal axis represents the left eye; an even number represents the right eye.

Cumulative frequency of fixation distance was smaller in MNL than in non-MNL in [0, 25], [0, 50], [0, 75], and [0, 100] pixels, and the largest difference was in [0, 75] pixels (63.60 ± 3.02 % vs. 82.53 ± 1.68 %, P < 0.001). Figure 3 shows Subject 1’s fixation trajectory in MNL (A) and non-MNL (B). The fixation positions were more concentrated in non-MNL.

#### Pupil size

Analysis of the adjusted pupil size revealed significantly smaller APS_trial_, APS_sac,_ and APS_fix_ in MNL than in non-MNL and the APS is asymmetric between left and right eye (Table 3). The percentage of APS in [55%, 100%] was significantly smaller in MNL than in non-MNL. Figure 3(C) shows subject 5’s cumulative frequency of APS in [55%, 100%]. Compared to MNL, the cumulative frequency of APS in [55%, 100%] was obviously higher in non-MNL. The maximum cumulative frequency of APS in MNL was observed at [45%, 50%] interval (the interval’s index is 10), and in non-MNL was at [65%, 70%] interval (the interval’s index is 14) (P < 0.001, Table 3).

**Table 2.** Saccade amplitude and fixation distance compared over MNL and non-MNL.

**Parameters**	**MNL**	**non-MNL**	**Diff. of Mean**	**P-value**
	***Mean ± SE***	***Mean ± SE***		
FixDis in [0,25] pixels (%)	29.05 ± 2.61	43.22 ± 2.33	-14.18	**< 0.001**
FixDis in [0,50] pixels (%)	48.70 ± 3.28	66.67 ± 2.22	-17.97	**< 0.001**
FixDis in [0,75] pixels (%)	63.60 ± 3.02	82.53 ± 1.68	**-18.92**	**< 0.001**
FixDis in [0,100] pixels (%)	75.80 ± 2.89	90.35 ± 1.22	-14.56	**< 0.001**
SacAmp (degrees)	2.48 ± 0.19	1.45 ± 0.08	**-**	**< 0.001**
SacAmp > 1.5° (%)	61.38 ± 2.98	44.13 ± 2.29	17.26	**< 0.001**
SacAmp > 2.0° (%)	54.22 ± 2.96	34.41 ± 2.17	19.82	**< 0.001**
SacAmp > 2.5° (%)	46.60 ± 2.96	25.12 ± 1.83	**21.48**	**< 0.001**
SacAmp > 3.0° (%)	39.26 ± 2.84	19.33 ± 1.62	19.93	**< 0.001**
SacAmp > 3.5° (%)	32.17 ± 2.66	14.08 ± 1.36	18.09	**< 0.001**
SacAmp > 4.0° (%)	25.33 ± 2.71	11.14 ± 1.16	14.19	**< 0.001**
SacAmp > 4.5° (%)	21.66 ± 2.37	8.36 ± 1.11	13.29	**< 0.001**
SacAmp > 5.0° (%)	18.78 ± 2.27	6.48 ± 0.95	12.30	**< 0.001**
SacAmp > 5.5° (%)	16.17 ± 2.01	5.01 ± 0.83	11.15	**< 0.001**
SacAmp > 6.0° (%)	12.99 ± 1.86	4.15 ± 0.81	8.84	**< 0.001**
SacAmp > 6.5° (%)	11.91 ± 1.73	3.65 ± 0.78	8.25	**< 0.001**
SacAmp > 7.0° (%)	11.03 ± 1.69	2.69 ± 0.64	8.33	**< 0.001**
SacAmp > 7.5° (%)	9.84 ± 1.62	2.03 ± 0.54	7.81	**< 0.001**

**Table 3.** Pupil size compared between MNL and non-MNL.

**Parameters**	**MNL**	**non-MNL**	**P-value**
	***Mean ± SE***	***Mean ± SE***	
APS of left eye in trial (%)	46.96 ± 1.54	64.38 ± 1.43	**< 0.001**
APS of right eye in trial (%)	46.99 ± 1.45	62.52 ± 1.60	**< 0.001**
APS of left eye in saccade (%)	46.22 ± 1.52	63.50 ± 1.49	**< 0.001**
APS of right eye in saccade (%)	46.80 ± 1.41	61.75 ± 1.63	**< 0.001**
APS of left eye in fixation (%)	47.61 ± 1.58	65.16 ± 1.39	**< 0.001**
APS of right eye in fixation (%)	47.28 ± 1.47	63.31 ± 1.60	**< 0.001**
cumulative frequency of APS in [55%,100%] (left eye) (%)	25.88 ± 4.03	72.07 ± 3.21	**< 0.001**
cumulative frequency of APS in [55%,100%] (right eye) (%)	26.53 ± 3.80	68.01 ± 3.45	**< 0.001**
index of maximum cumulative frequency of APS (left eye)	10.05 ± 0.33	13.71 ± 0.31	**< 0.001**
index of maximum cumulative frequency of APS (right eye)	9.93 ± 0.33	13.43 ± 0.36	**< 0.001**

### Intelligent Classification

#### Feature selection

Based on the significant statistical tests mentioned above, we chose the measures for feature selection. Specifically, the selected features included 4 significantly different time and frequency measures in Table 1 (MNL & non-MNL duration, saccade duration, and saccade/fixation number), 3 significantly different gaze traveling measures in Table 2 (cumulative frequency of fixation distance in [0, 75] pixels, saccadic amplitude, cumulative frequency of saccadic amplitude above 2.5 degrees), and 5 significantly different pupil adjustment measures in Table 3 (APS_trial_, APS_sac,_ APS_fix_, cumulative frequency of APS in [55%, 100%], and index of maximum cumulative frequency of APS).

#### Validity of synthesized data points

**Figure 4.**
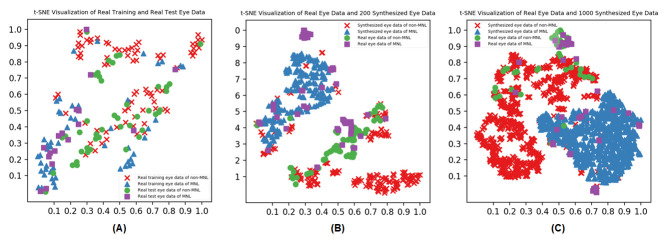
Verification of synthesized to the real human eye data by t-SNE-based visualization.

Figure 4 shows the visualization of real eye data (A), real eye data and 200 randomly selected synthesized eye data (B), real eye data and 1000 synthesized eye data (C) over three different data feeding strategies reported by t-SNE. The synthesized eye data overlapped with the same class of real eye data. There is a similar pattern between synthesized and real eye data.

#### MNL and non-MNL classification

**Figure 5.**
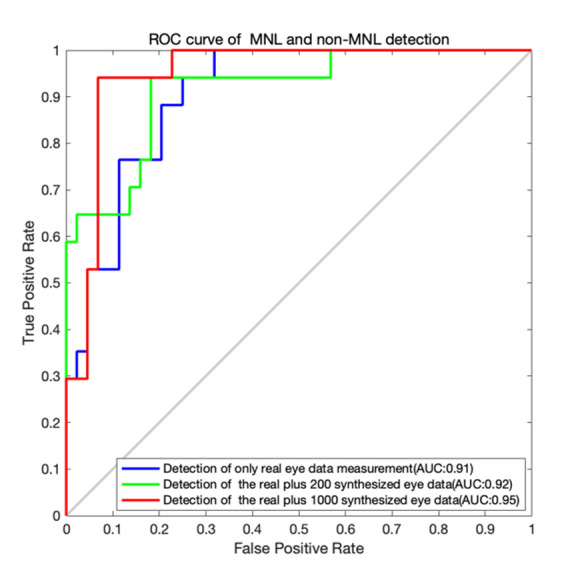
ROC curves for MNL and non-MNL classification.

The classification outputs by the LSTM algorithm are shown in Table 4 and the ROC curves for the MNL and non-MNL classification are shown in Figure 5. A training set of only the real data did not yield adequate classification outcomes. The accuracy, sensitivity, and specificity are moderate. When we included 200 synthesized data points for training, the outcome was improved. With a training set of 1000 synthesized data points, the detection accuracy, sensitivity, and specificity improved dramatically. The accuracy (91.80%), the sensitivity (90.91%), and specificity (94.12%) were both well. When we added more synthesized data points (1600, 2000), the performance of the LSTM algorithm declined (Table 4). So, the acceptable option is the real and 1000 synthesized eye data.

**Table 4.** MNL and non-MNL classification results. R represents real eye data; S represents synthesized eye data.

Training Set	Test Set	Accuracy	Sensitivity	Specificity
MNL: 51 (R) non-MNL: 77(R)	MNL: 17(R) non-MNL: 44(R)	81.96%	79.55%	88.24%
MNL:51(R)+200(S) non-MNL: 77(R)+200(S)		83.61%	79.55%	94.12%
MNL: 51(R)+1000(S) non-MNL: 77(R)+1000(S)		91.80%	90.91%	94.12%
MNL: 51(R)+1600(S) non-MNL: 77(R)+1600(S)		88.52%	95.45%	70.59%
MNL: 51(R)+2000(S) non-MNL: 77(R)+2000(S)		83.61%	86.36%	76.47%

## Discussion

We are delighted that the deep learning algorithm can identify the moment of task difficulty from eye-tracking data recorded in multiple long colonoscopic procedures. Colonoscopic videos were only used for verifying the outcomes. The eye-tracking provides rich and continuous streams of data for identifying different behaviors between the MNL and non-MNL.

In this study, we took a step to optimize the feeding data by pre-selecting key features for deep learning. Specifically, we screened several features and selected those displaying significant differences between the different statuses of navigation (MNLs or non-MNLs). Contradicting our expectation, performers’ pupil size was smaller in MNL than in non-MNL. The exact reason behind this finding is unknown to us. Graphic scenes during non-MNL often included darker areas (lumen and surrounding structure than scenes in MNL (Figure 1), which may be a reason for the larger pupil dilation in non-MNL. In addition, performers in non-MNL might be more engaged in the colonoscopic tasks as they vigorously manipulated the scope to move it forward. They might also need to enlarge their pupil to inspect the interior GI structure to find polyps or other abnormalities. Increasing pieces of evidence have shown that pupil dilation is affected by how visual input is collected, processed and used for guiding the movement (19). Nevertheless, the pupil still displayed a difference between normal and poor performance, providing a source of data to train the computer algorithm for classifying behaviors in colonoscopy.

Once we selected the appropriate set of feeding data, we need to increase the volume of the data for deep learning. In reality, it is difficult for us to recruit a large group of participants from a single health organization. It is not uncommon that deep learning algorithms have to run on data with a relatively small sample size. To compensate for the small volume of real data, we introduced the DCGANs to learn characteristics of real human data, then generated a new set of data.

Regardless of the type of algorithms used for data synthesis, a validity checking on the synthesized data points is necessary (6; 26). In this study, data synthesized by the DCGANs were augmented nicely to real human data (Figure 4), which constructed a sound foundation for later data classification. The total number of synthesized data points used for deep learning also needs to be carefully determined. In our practice, feeding 1000 synthesized data points dramatically increased the accuracy (91.80%), sensitivities (90.91%), and specificity (94.12%). Adding 1000 synthesized data points to the human data produced a balanced outcome (Table 4).

Past researches on eye-tracking have proved that eye signals can report performers’ visual searching strategies (11), eye-hand coordination patterns, and workload levels (3; 12). Our goal is to develop an AI-based teaching platform for training healthcare skills with minimal engagement from faculty members. This teaching system will detect the moment of trainee’s task difficulty and provide an instructional message to a trainee when needed. We are glad to see our current research demonstrate that the eye-tracking signals are sufficient to identify the moment of navigation loss. Computer outputs are matched to experts’ judgment. Eye-tracking data enable us to rapidly examine the performance and spontaneously report those behaviors that connect to trainees’ performance during colonoscopy.

This study focuses on one aspect of trainees’ behaviors (i.e., navigation loss) that connects to their task difficulty during endoscopy. The achievement of this study is not the endpoint of our research as trainees’ task difficulties are multifarious. By closely working with endoscopists, we will extend our research to inspect more behaviors during endoscopic procedures.

There are some limitations to this study. First, eye-tracking data was collected from a colonoscopic simulator. Caution is needed when applying our results to clinical scenarios. Second, the volume of real human data was relatively small. We expect to see an improved outcome when we enlarge our sample size in future studies. Third, navigation loss in any colonoscopy is a sign of incompetent practice, but there are other behavioral markers for describing the skill level of the performers. Moreover, we notice the limitation in calculating pupil responses when the sampling frequency of eye-tracking is low. We plan to include more behavioral indicators in the future for detecting the moment of performance difficulty, such as navigation losing, scope withdrawing (zoom-out), and incorrect angulation movement. Knowledge gained from this series of studies makes a steadily forward step to our research goal, but we still have a way to go before we can confidently design an education system using AI technology.

## Conclusions

In conclusion, a series of specific indicators on eye gaze pattern and pupillary response on the MNL was founded; the real measures displaying significant differences with 1000 synthesized data points generated a better outcome by the deep learning algorithms, which helped us to identify the moment of task difficulty during colonoscopy. This project is the first step to our goal of creating an intelligent skill training system where it can automatically detect the task difficulty and deliver appropriate instructional messages at the right moments. We believe the same AI approach can be further applied to inspect target behaviors from other healthcare procedures.

### Ethics and Conflict of Interest

The author(s) declare(s) that the contents of the article are in agreement with the ethics described in http://biblio.unibe.ch/portale/elibrary/BOP/jemr/ethics.html and that there is no conflict of interest regarding the publication of this paper.

### Acknowledgements

This research was supported in part by grant RGPIN-2016-06462 from the Natural Sciences and Engineering Research Council of Canada (NSERC), in part by grant UOFAB TLEF RES0030076 from the University of Alberta Provost Office, in part by grant 61801019 from the National Natural Science Foundation of China, in part by grant 201906465021 from the China Scholarship Council, in part by grant FRF-DF-20-04 and FRF-BD-19-012A from the Fundamental Research Funds for the Central Universities.
